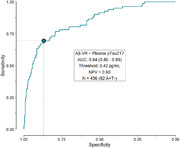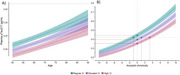# Effect of Corpulence on pTau217 Measurements in Plasma

**DOI:** 10.1002/alz70856_101237

**Published:** 2025-12-24

**Authors:** Ramiro Eduardo Rea Reyes, Rachael E. Wilson, Julie E. Oomens, Rachel L Studer, Elysse Keske, Aaron Fredricks, Martie Marshall, Cindy Jensen, Monica VandenLangenberg, Beckie Jeffers, Erin M. Jonaitis, Rebecca E. Langhough, Nathaniel A. Chin, Sanjay Asthana, Henrik Zetterberg, Sterling C Johnson

**Affiliations:** ^1^ Wisconsin Alzheimer's Disease Research Center, Madison, WI, USA; ^2^ Wisconsin Alzheimer's Disease Research Center, University of Wisconsin‐Madison, School of Medicine and Public Health, Madison, WI, USA; ^3^ Wisconsin Alzheimer's Disease Research Center, University of Wisconsin School of Medicine and Public Health, Madison, WI, USA; ^4^ Wisconsin Alzheimer's Institute, University of Wisconsin‐Madison School of Medicine and Public Health, Madison, WI, USA; ^5^ University of Wisconsin‐Madison, Madison, WI, USA; ^6^ Wisconsin Alzheimer's Institute, University of Wisconsin School of Medicine and Public Health, Madison, WI, USA; ^7^ Wisconsin Alzheimer's Disease Research Center, University of Wisconsin School of Medicine and Public Health, Madison, WI, USA; ^8^ University of Wisconsin School of Medicine and Public Health, Madison, WI, USA; ^9^ Hong Kong Center for Neurodegenerative Diseases, Hong Kong, Science Park, China; ^10^ Department of Neurodegenerative Disease, UCL Institute of Neurology, London, United Kingdom; ^11^ Clinical Neurochemistry Laboratory, Sahlgrenska University Hospital, Gothenburg, Sweden; ^12^ UK Dementia Research Institute at UCL, London, United Kingdom; ^13^ Department of Psychiatry and Neurochemistry, Institute of Neuroscience and Physiology, The Sahlgrenska Academy, University of Gothenburg, Mölndal, Sweden; ^14^ Wisconsin Alzheimer's Disease Research Center, School of Medicine and Public Health, University of Wisconsin‐Madison, Madison, WI, USA; ^15^ Wisconsin Alzheimer's Institute, University of Wisconsin School of Medicine and Public Health, Madison, WI, USA

## Abstract

**Background:**

Plasma pTau217 is a robust indicator of amyloid pathology recently included as a Core 1 biomarker for Alzheimer's disease (AD). However, factors like body mass can influence its measurement. Investigation of how much this impacts classification of participants as Core 1 A+ is relevant for studies looking at early AD stages. In this work, we assess the effect of corpulence on concentrations of plasma pTau217 and other biomarkers.

**Method:**

We analyzed longitudinal plasma samples from 746 participants in the Wisconsin Registry for Alzheimer's Prevention and the Wisconsin Alzheimer's Disease Research Center with recent PET‐PiB and PET‐MK6240 data (max 1.5 years before last plasma sample). We measured five blood biomarkers (pTau217‐ALZpath, GFAP, NfL, Aβ_40_, and Aβ_42_) using the Quanterix HD‐X platform. Amyloid onset age was estimated using the sampled iterative local approximation algorithm (SILA) on PET‐PiB uptake values (locally‐derived Amyloid+ threshold ∼17 Centiloids). We modeled biomarker levels using mixed gamma regression; terms included age, corpulence, APOEε4 carriage, and sex. For pTau217, we modeled amyloid non‐accumulators and accumulators separately, using SILA‐chronicity instead of age in the latter. An ROC analysis was used to assess pTau217 classification performance against PET‐PiB visual reads on the subset that was also tau negative via PET‐MK6240 visual read.

**Result:**

Most participants were cognitively unimpaired at first plasma sample. (697, 93.4%; 511 females; 39.4‐93.8 yo, mean(SD) age=65.7(7.7)). The ROC‐determined pTau217 threshold was 0.42 pg/mL (Figure 1; AUC(CI)=0.84 (0.8‐0.89). Everypoint increase in corpulence (centered at 13, regular corpulence) was associated with a 4‐5% reduction in pTau217 levels in both amyloid accumulators (β(CI)=0.95(0.92–0.97), *p* <.001) and non‐accumulators (β(CI)=0.96(0.94‐0.98), *p* <.001). For amyloid accumulators, this represents ∼3.25 years of delay to reach the ROC threshold among individuals with obesity (corpulence=21). This reduction was also present in concentrations of GFAP (β=0.96, CI=0.94‐0.97, *p* <.001) and NfL (β=0.96, CI=0.94‐0.97, *p* <.001) but not detected in the Aβ_42_/Aβ_40_ ratio (*p* = .550).

**Conclusion:**

Our results show that corpulence, a body mass measure, is associated with plasma biomarker levels. Accounting for corpulence might lead to significant improvements in plasma‐based early detection of AD.